# Efficacy of Autologous Conditioned Serum on the Dorsal Root Ganglion in Patients with Chronic Radicular Pain: Prospective Randomized Placebo-Controlled Double Blind Clinical Trial (RADISAC Trial)

**DOI:** 10.3390/jcm14217771

**Published:** 2025-11-01

**Authors:** Marta Homs, Raimon Milà, Jordi Recasens, Diego Delgado, Rosa Maria Borràs, Ricard Valdés, David Parés

**Affiliations:** 1 Department of Anesthesiology and Pain Medicine, Dexeus University Hospital, 08028 Barcelona, Spain; 2 School of Medicine, Universitat Autònoma de Barcelona, 08193 Bellaterra, Spain; 3 Blanquerna Faculty of Health Sciences, Ramon Llull University, 08022 Barcelona, Spain; 4 Advanced Biological Therapy Unit, MiKS Hospital, 01010 Vitoria-Gasteiz, Spain; 5 General Surgery Department, Germans Trias i Pujol University Hospital, 08916 Badalona, Spain

**Keywords:** lower limb radicular pain, lumbar radicular pain, autologous conditioned serum, pulsed radiofrequency, dorsal root ganglion

## Abstract

**Background**: Pulsed radiofrequency (PRF) applied to the dorsal root ganglion (DRG) has been proposed as an effective neuromodulator treatment for persistent radicular pain. Autologous conditioned serum (ACS) therapy, derived from the patient’s own blood, offers a conservative approach. This study aims to evaluate the efficacy of ACS applied to the DRG as an adjunct in treating lower limb radicular pain (LLRP). **Methods**: A prospective, randomized, double-blind, placebo-controlled clinical trial was conducted comparing PRF combined with ACS versus PRF with physiological saline (PhS) on the DRG. Seventy patients (35 per group) with radicular pain lasting ≥6 months and refractory to previous treatments were enrolled. The primary outcome measure was the Numeric Pain Rating Scale (NPRS); secondary measures included the Oswestry Disability Index (ODI), Mood Assessment Scale (MOAS), SF-12 quality of life questionnaire, and DN4 neuropathic pain scale. Assessments occurred at baseline, 1 month, 3 months, 6 months, and 12 months post-intervention. **Results**: A total of 70 patients were included. The ACS group showed a significant reduction in pain compared to controls at 30 days (*p* < 0.05). Additionally, neuropathic symptoms such as tingling, numbness, stubbing, and burning decreased significantly in the ACS group during this period (*p* < 0.05). While both groups experienced pain reduction over time, no significant differences persisted at 6 months. No adverse effects were reported. **Conclusions**: The addition of ACS to PRF provides a short-term, statistically significant reduction in radicular pain at 30 days, suggesting it is a safe and effective adjunct therapy for lower limb radicular pain.

## 1. Introduction

Lower limb radicular pain (LLRP) is a common and costly cause of disability, significantly impairing patients’ daily functioning. It manifests as pain radiating to an extremity or trunk due to activation of nociceptive fibers originating from spinal nerves, often associated with lesions in the dorsal root ganglion (DRG) or spinal nerves, leading to ischemia or inflammation [1,2]. The annual prevalence ranges from 9.9% to 25%, classifying LLRP as a prevalent form of neuropathic pain [3,4]. While approximately 70% of patients experience symptom resolution within 12 weeks, 20–30% develop persistent pain beyond three months, adversely affecting quality of life and prognosis [5]. Chronic neuropathic pain impacts multiple aspects of health, transitioning from a symptom to a distinct disease entity [6,7]. Treatment options include pharmacotherapy, physiotherapy, and interventional procedures such as epidural injections and surgery; however, outcomes are often inconsistent [8,9,10,11,12]. A deeper understanding of the neurohistological and neurochemical mechanisms underlying radicular pain is essential for developing effective therapies, with the DRG serving as a key target for neuromodulation strategies [13]. Glial activation and inflammatory mediators contribute to sensitization processes, while hyperexcitability in afferent fibers—linked to alterations in gene expression and ion channel function—leads to spontaneous discharges characteristic of neuropathic pain [14,15,16].

Pulsed radiofrequency (PRF) targeting the DRG has emerged as a promising neuromodulator intervention for persistent radicular pain [17,18]. Its efficacy is attributed to its ability to modulate neuronal activity without causing significant tissue damage, making it minimally invasive, cost-effective, and associated with low complication rates [19,20,21,22]. PRF exerts analgesic effects through mechanisms involving thermal energy generation and electrical fields that alter DRG neuron excitability [23,24]. Evidence suggests that PRF can produce substantial pain relief with benefits lasting up to one year in some cases [24,25,26,27]. However, the evidence for this treatment in patients with LLRP is still low and moderate, and its efficacy with respect to other treatments such as exercise or corticosteroids is limited, especially in the long term [28]. Therefore, the association of other treatments that act synergistically with the mechanisms of action of PRF may be a good choice for the improvement of this treatment.

In this regard, biological therapies have gained increasing attention. Autologous conditioned serum (ACS) involves incubating the patient’s own blood to produce serum rich in a large number of biomolecules. These include growth factors and anti-inflammatory cytokines such as interleukin-1 receptor antagonist (IL-1Ra), anti-inflammatory cytokines (IL-4, IL-10, and IL-13) [29,30]. The therapeutic potential of this biological product, in addition to its biosafety, is based on reducing inflammation and promoting tissue repair [31,32]. Administered via multiple injections, ACS has demonstrated efficacy in reducing pain and improving function and quality of life without significant adverse effects compared to corticosteroids [33,34,35,36].

Bearing all this in mind, the objective of this study is to evaluate the efficacy and safety of adding ASC application to PRF treatment on neuropathic pain in patients with persistent LLRP. The hypothesis is based on the fact that addressing both the electromagnetic and neurochemical pathways that influence DRG not only alleviates symptoms but also reverses the neuronal dysfunction associated with chronic neuropathic conditions.

## 2. Materials and Methods

### 2.1. Study Design

This study is a prospective, double-blind, randomized, placebo-controlled trial aimed at evaluating the efficacy of PRF combined with ACS administered to the DRG in comparison to PRF combined with 0.9% Physiological Saline (PhS) for the reduction in neuropathic pain and its associated consequences in patients suffering from persistent LLRP.

It was carried out in accordance with the international standard on clinical trials: Real Decreto 223/2004, Declaration of Helsinki in its latest revised version (Fortaleza, Brazil; 2013), Good Clinical Practice Regulations (International Conference for Harmonization), and the CONSORT guidelines [37,38]. The study received approval from the Committee on Ethics and Drug Research (CEIm) of the hospital group QuironSalud-Catalunya and was registered with the European Agency for Clinical Trials (EudraCT number: 2021-005124-38) (18 November 2021). All patients provided written informed consent before entry into the clinical trial.

### 2.2. Study Population

The research was conducted at the Pain Unit within the Department of Anesthesiology, Resuscitation, and Pain Management at Dexeus University Hospital (DUH) in Barcelona from November 2021 to September 2024. The target population consists of 70 patients aged over 18 years who are literate and have experienced radicular pain in the lower limb for more than six months. Symptomatic diagnostic clinical screening criteria will be employed to confirm LLRP prior to patient enrolment. Inclusion and exclusion criteria are detailed in Table 1 as well as the RADISAC Protocol [39].

### 2.3. Preparation of ACS

All patients had 20 mL of whole blood drawn using a special syringe with an increased inner surface area (Orthogen, Düsseldorf, Germany). Simultaneously, tests were conducted for human immunodeficiency virus (HIV), syphilis, hepatitis B, and hepatitis C. Medical-grade glass beads within the special syringes increase the nonpyrogenic surface area. These glass spheres induce dose-dependent production of IL-1Ra by white blood cells in whole blood incubated at 37 °C. After 6 h of incubation, the blood-filled syringes were centrifuged at 4000 rpm for 15 min, and the serum supernatant was aliquoted into two 4 mL syringes. The aliquots were then frozen at −20 °C. On the day of injection, the syringes were taken out of the freezer prior to administration.

In patients of the PhS group, 10 mL of blood was drawn into a blood bag covered with an opaque wrapper similar to the ACS group to keep the patients blinded.

### 2.4. Treatment Administration

Randomization was executed using a simple randomization method based on an equiprobable algorithm. Both the principal investigator and participants remained blinded throughout the study.

**Figure 1:**
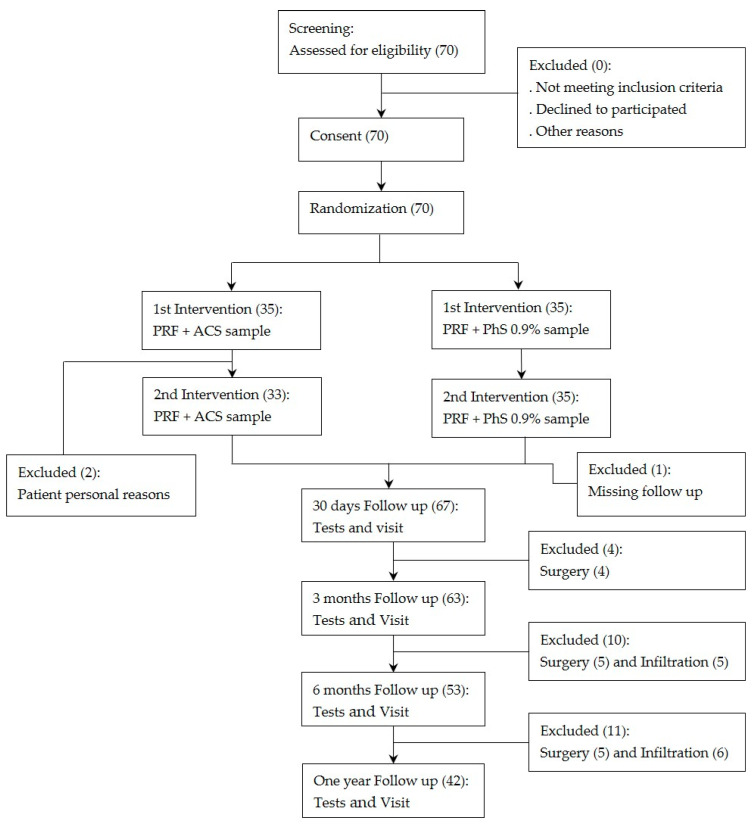
The patient flow diagram of the study. PRF: Pulsed radiofrequency; ACS: Autologous conditioned serum.

The intervention protocol was designed to apply PRF therapy to the DRG for 8 min at 45 V in the affected root for all patients included in the study. Upon completion of PRF therapy, a 3 mL dose of ACS was administered to the DRG in patients in the treatment group, whereas patients in the placebo group received a 3 mL dose of 0.9% FSS. Based on the existing literature regarding ACS therapy, the study protocol also included a second administration of ACS or saline solution 15 days after the first intervention, following the same procedural protocol (Figure 1: Flow Diagram)

**Figure 2:**
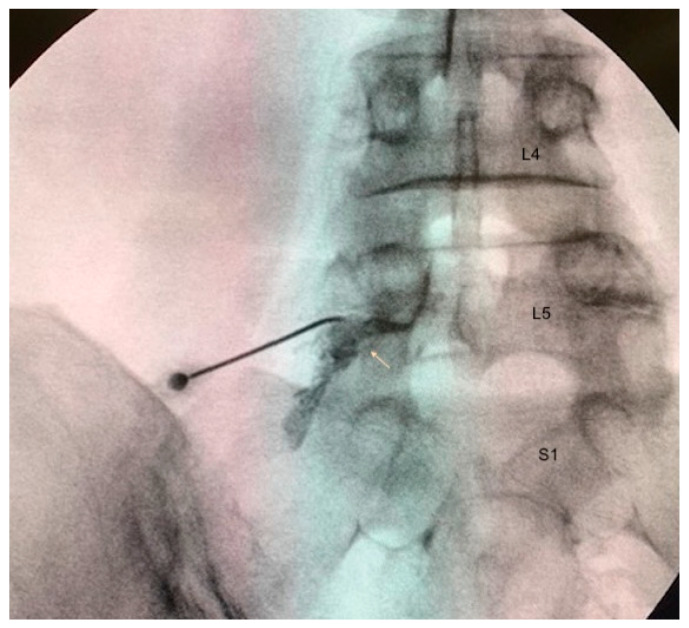
Fluoroscopic image of the procedure. The arrow in the image depicts the localization of the dorsal root ganglion (DRG) within the foramen, outlined by ionic contrast. The L4, L5, and S1 vertebrae are anatomically marked according to the fluoroscopy image.

On the day scheduled for infiltration, proper patient positioning and monitoring according to Pain Unit protocols were essential prior to performing the procedure. A transforaminal approach to access the DRG was utilized under aseptic conditions and fluoroscopic guidance. After verifying impedances (not exceeding 450 ohms), sensory stimulation was assessed using voltages between 0.3 and 0.6 V, while motor stimulation required a voltage sufficient to elicit paresthesia—typically no more than double that required for sensory stimulation. Following confirmation via iodinated contrast injection, PRF therapy will be administered to the DRG (8 min at 45 V) (Figure 2). Upon completion of this therapy, participants received their assigned treatment. Patients assigned to the control group received 3 mL of 0.9% PhS, and those assigned to the experimental group were administered 3 mL of ACS. Fluoroscopic-guided injections were performed directly into the DRG.

### 2.5. Clinical Outcomes

The primary outcome measure was pain level assessed using the Numeric Pain Rating Scale (NPRS), which allowed respondents to select a whole number (0–10) reflecting their pain intensity. A reduction of ≥2 points on this scale is considered clinically significant relief. This outcome was measured at baseline and again after treatments at 30 days, 3 months, 6 months, and 12 months post-intervention [40,41,42].

Secondary outcomes include Oswestry Low Back Pain Disability Index (ODI) [42,43]; Mood Assessment Scale (MOAS) or Mood Rating Scale (MRS) [44]; Quality of Life assessment using SF-12 [45,46]; and *Douleur Neuropathique en 4* questions (DN4) [47,48]. These measures were also collected at baseline as well as at one month, three months, six months, and twelve months following intervention.

The Oswestry Low Back Pain Disability Index (ODI) is a 10-item questionnaire designed to assess functional disability associated with low back pain. Each item is scored from 0 to 5 and expressed as a percentage (0–100%), with higher scores indicating greater functional limitation.

The Mood Assessment Scale (MOAS) or Mood Rating Scale (MRS) is a self-report instrument that evaluates mood status, including domains such as sadness, anxiety, and irritability, as well as their impact on daily functioning. Higher scores reflect greater affective disturbance and allow monitoring of changes following intervention.

The Short Form-12 (SF-12) is a 12-item questionnaire that yields two summary components: the Physical Component Summary (PCS) and the Mental Component Summary (MCS), both standardized to a mean of 50 (higher scores indicate better outcomes). It assesses perceived health status and health-related quality of life.

The *Douleur Neuropathique en 4* questions (DN4) is a screening tool for neuropathic pain composed of 10 items, including seven symptom-related and three clinical examination items. A total score of ≥4 suggests the presence of neuropathic pain, with good sensitivity and specificity.

At baseline, demographic data regarding patients’ previous failed therapies—including frequency, duration, type, and compliance—as well as results from MRI and EMG diagnostic tests were documented. Analgesic use for LLRP treatment—including frequency, dosage, and duration—was also recorded. All baseline assessments and follow-up evaluations involved collecting reported outcomes through completion of NPRS, ODI, MOAS, SF-12, and DN4 questionnaires alongside documentation of narcotic and non-narcotic analgesic usage.

### 2.6. Safety Outcomes

All adverse events related to study participation were monitored and reported comprehensively—including severity assessment—and their relationship to study devices/procedures determined by investigators as either related specifically or classified as serious adverse events.

Device-related adverse events, along with all serious adverse events occurring throughout the study period, were documented by the main investigator of the trial.

### 2.7. Sample Size Calculation

Power analysis was conducted to estimate the minimum sample size needed to achieve 80% power at a 5% level of significance for the primary outcome measures. An effect size of 2 points with a standard deviation (SD) of 2.8 points was assumed. This analysis suggested that a minimum of 35 patients per group would be required, expecting a dropout rate of 0.1.

### 2.8. Statistical Analysis

Descriptive statistics were computed for all relevant variables. Means, standard deviations, and confidence intervals (95%) were calculated for the dependent variables. The normality of the distribution was assessed using the Kolmogorov test, skewness, kurtosis, and visual inspection of histograms. No major violation of normality was observed. For categorical variables, frequencies and percentages were reported. Longitudinal data were analyzed using a linear mixed-effects model to examine changes in the dependent variable over time points (30 days, 3 months, and 6 months). Time was included as a fixed effect, and individual participants were treated as random effects to account for the repeated-measure structure of the data. The model included group as a fixed between-subject factor, and the time x group interaction was tested to examine differential changes over time. An unstructured covariance matrix was used to model within-subject correlations across time points, allowing for unequal variances and covariances. The model was estimated using restricted maximum likelihood (REML). Participants’ ages were included as a covariate to control for individual differences in baseline performance. Incidence rates for complications were evaluated using chi-square tests. Statistical significance was set at *p*-value < 0.05 (two-tailed). All analyses were performed using IBM SPSS Statistics (version 25).

## 3. Results

### 3.1. Patient Characteristics

Figure 1 depicts the flow diagram of the patients in the study. A total of 70 patients were eligible and included in the study. There were 67 patients who completed follow-ups over the period of 30 days. 63 patients for follow-ups over the period of 3 months and 53 patients for follow-ups of 6 months. At 12 months, only 42 patients of the initial 70 completed the 12-month follow-up; hence, this time point was not included in the final analysis.

Table 2 shows patient characteristics according to demographic and clinical variables. Most patients experienced radicular pain, with 91.2% in the experimental group and 94.4% in the control group, respectively. There was no statistically significant difference in age and gender between both groups, kind of pain, affected extremity, consumption of analgesics or neuromodulators, or even previous treatments (*p* > 0.05).

### 3.2. Clinical Evaluation

**Figure 3:**
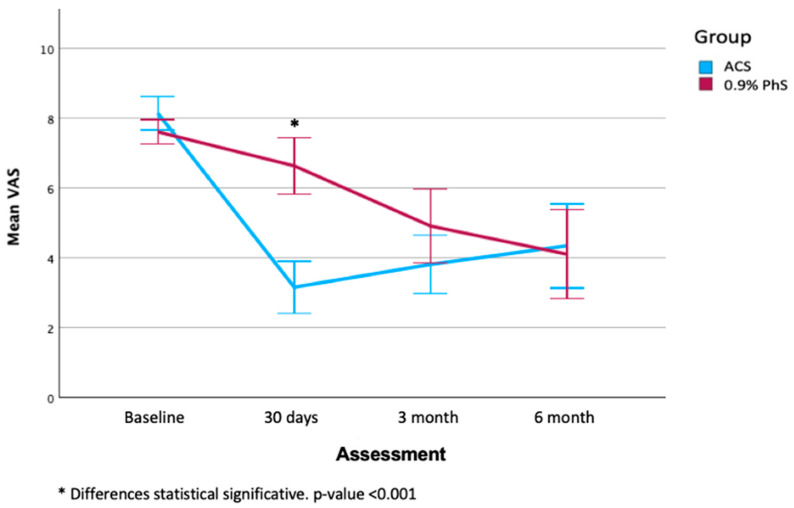
The graph demonstrates a reduction in pain in both groups, with statistical significance observed during the initial period.

Table 3 shows the values of both groups across the evaluated periods (baseline, 30 days, 3 months, and 6 months). No statistically significant differences were found in the basal period to NPRS, DN4, Oswestry scale, and MOAS (*p* > 0.05). According to the follow-up, there was a statistically significant improvement in the values of the VAS in both groups throughout the three time points (*p* = 0.003). A significant improvement was also observed in both groups at the end of the 6-month follow-up. It was observed that the improvement over time in both groups does not reach statistical significance when directly comparing the two groups (*p* = 0.194). However, analysis of the initial period (30 days) indicated a statistically significant difference in the NPRS scores, favoring the experimental group versus the control group (3.16 ± 2.02 vs. 6.64 ± 2.37, respectively, *p* < 0.001) (Figure 3).

For the DN4 variable, although pain decreased in both groups, this reduction was not statistically significant (*p*-value = 0.292); similarly, no significant difference between groups over time was found (*p*-value = 0.066). Regarding the MOAS variables, significant improvements were noted across all dimensions (depression, anxiety, anger, and happiness) throughout the study (*p*-value < 0.05); however, as with previous variables, no significant differences between groups were observed during follow-up (*p*-value > 0.05) (Table S1 (Supplementary Material)).

For the Oswestry Disability Index, a significant improvement over time was seen in both groups. The experimental group experienced a change of approximately 10 points on the scale from baseline to the end of the 6-month follow-up (from 23.41 ± 10.49 to 14.04 ± 9.46); similarly, the control group showed a comparable reduction (from 21.47 ± 10.49 to 13.64 ± 10.48 points) (*p*-value < 0.001) (Table S1, Supplementary Material).

**Figure 4:**
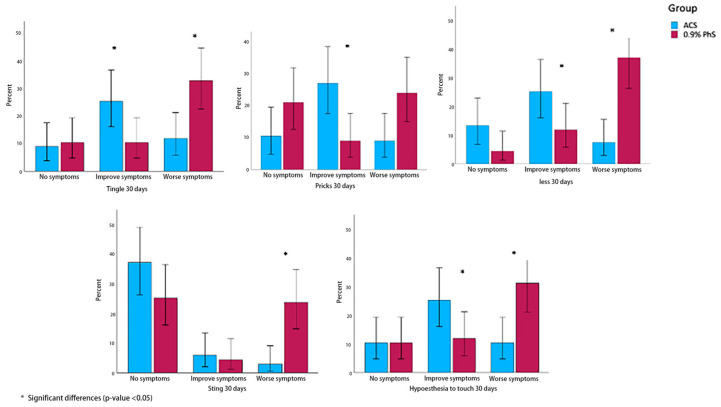
Graphic of both groups of DN4 symptoms at 30 days: Tingle, Pricks, Numbness, Sting, Hypoesthesia to touch.

Table 3 shows that symptoms such as tingling (54.8%, 0.005), stabbing sensations (58.1%, *p* = 0.002), numbness (54.8%, *p* > 0.001), burning sensation (12.9%, *p* = 0.002), and hypoesthesia (54.8%, *p* = 0.007), exhibited a statistically significant improvement at 30 days relative to the control group. For the remaining variables, a trend toward improvement is observed; however, these differences do not reach statistical significance (Table 3, Figure 4). This improvement stabilized in both groups over the 3- and 6-month periods, with no statistically significant differences observed between them (*p* > 0.05).

### 3.3. Safety Evaluation

No complications or adverse effects were reported after the fluoroscopy-guided PRF on DRG and posterior transforaminal injection of SAC or 0.9% PhS during the 1-year follow-up period. The CONSORT checklist is included as Supplementary Material accompanying this manuscript [38].

## 4. Discussion

The main finding of this study was that the application of repeated ACS injections together with PRF provides a significant improvement in short-term pain compared to the application of PRF with saline. Moreover, this study also demonstrated improvements in pain, functionality, and quality of life in both treatment groups, suggesting that PRF is a treatment that provides long-term pain relief.

These results are in agreement with the results of several studies confirming that the PRF applied to the DRG presents antinociceptive effects in lower limb radiculopathy [20,21,22,23,24,25,26,27]. Unlike work conducted by Park et al., this study also provides Level I evidence that pulsed radiofrequency on the DRG is an effective and safe treatment for patients with LLR regarding pain and function [28].

When interpreting the results, several points should be considered; firstly, considering the ACS group, a statistically significant improvement was observed during the initial period, indicating that ACS likely has a very good short-term effect comparable, for example, to corticosteroids [49,50]. However, when considering other reviews or comparative studies of platelet-rich plasma (PRP) with Cs—none of which included pulsed radiofrequency—the current study did not observe a long-term effect demonstrating statistically significant superiority.

A possible explanation for a short-term improvement in pain compared to the control group could lie in the application protocol, more specifically, in the number of injections. Becker et al. reported significant pain reduction lasting up to six months with three doses of ACS [49]. In a study conducted by Godek et al. the authors applied up to six doses of ACS to the foraminal space, observing significant pain reduction up to three months [51]. Other work involving 20 patients who received three injections (without a control group) showed significant improvement up to six months [52]. In contrast, a pilot study by Goni et al. found improvement only with a single dose of ACS in comparison to methylprednisolone. After administering ACS versus methylprednisolone at the foraminal level, they observed equivalent improvements in both groups, with the effect persisting longer in the ACS group. However, the pathology under study was cervical radiculopathy, which could also affect the results of these treatments [53]. Thus, the results suggest that adding a third dose of ACS, or more, might potentially produce statistically significant pain relief at three and six months, according to previous studies. Additionally, there was a statistically significant reduction in DN4 symptoms during the first period, which could reflect a neuromodulator effect of neuropathic pain by ACS, as described in similar effects in other studies [49,54,55].

This has clinical relevance, as current neuropathic pain guidelines do not include regenerative medicine in the standard treatment options [15]. The treatment of neuropathic pain with regenerative medicine, including peripheral nerve or dorsal root treatment using autologous blood derivatives, currently has limited evidence in the existing literature. Nonetheless, several lines of evidence suggest that blood-derived products offer numerous benefits in neuronal damage regeneration and distal function. This is often accompanied by an improvement in symptoms [56,57]. Similar findings have been reported in other studies involving conditions such as diabetes mellitus [55], trigeminal neuralgia [54], leprosy [58], and peripheral nerve injury [59], where the use of blood-derived products was associated with a reduction in neuropathic pain and potentially contributed to nerve regeneration [60,61].

Secondly, a review of the literature suggests that molecular neuromodulation in ACS can be explained by its bioactive molecular content. ACS contains high concentrations of cytokines such as IL-4, IL-10, IL-13, and IL-1 receptor antagonist (IL-1Ra), as well as growth factors like FGF-2 and TGF-β1. Notably, IL-1Ra functions as a biochemical sensitizer of nerve roots in radiculopathy [62]. Interleukin-1 (IL-1) is particularly significant among cytokines involved in orthopedic diseases [30,63]. Multiple studies emphasize that these cytokines play a fundamental role in modulating neuropathic pain symptoms, contributing to the pathophysiology and potential therapeutic targets of pain management [64].

Thirdly, from a broader perspective within regenerative medicine, recent systematic reviews [65,66,67] on lumbar/radicular pain and regenerative medicine have found insufficient evidence regarding the use of blood-derived products for radicular pain management. Only 12 studies are highlighted—three randomized controlled trials—evaluating autologous serum/PRP administered via epidural or transforaminal routes. Comparative groups used corticosteroids or local anesthetics; some studies did not include PRF therapy on the DRG. Four studies also treated disk pathology, facet joints, or zygapophyseal joints concurrently. Despite these limitations, conclusions favor biological therapies over corticosteroids for radicular pain due to their longer-lasting effects beyond 6–12 months—a finding differing from our current results [65,66,67].

Although long-term differences between groups were not statistically significant, it is plausible that a synergistic effect exists between the neuromodulation provided by ACS and PRF. This interaction was previously described by Jorge et al. concerning neuropathic pain control and neuromodulation, although in this study, no synergistic effect between the groups was observed [68].

Finally, regarding safety, this clinical trial clearly demonstrates an advantage for ACS treatment since no complications or adverse effects were observed in any case. This aligns with several works, which indicated that regenerative medicine treatments at the axial level have a very safe therapeutic profile in terms of adverse effects and side effects, in contrast to corticosteroid treatments [66,67].

One of the limitations of this study is the final analysis period, which was excluded from the results due to n-data loss. Since radicular pain is a highly functionally limiting type of neuropathic pain, patients who required additional treatment or surgery were excluded from the analysis, thereby reducing the sample size at the 12-month follow-up and consequently decreasing the statistical power of the findings. In the same line, improvement was observed at the 3- and 6-month follow-up periods; however, this improvement was not statistically significant in favor of ACS. A possible explanation for this finding may lie in the insufficient sample size, considering the potential attrition observed during the 3–6-month periods. In addition, another limitation of the study is the lack of characterization of ACS, which could have provided more information on the mechanisms of action of this treatment and its influence on the results. However, due to the complexity of this product, its characterization is more complex and costly than other blood products such as PRP, requiring molecular analyses that will be taken into consideration in future studies. Finally, objective evaluations such as imaging studies or electromyograms in which disease modifications could be observed would provide a better understanding of the mechanics of these treatments.

Regarding clinical applicability, this study provides a strong level of evidence supporting the extrapolation of the proposed treatment to routine clinical practice. Incorporating regenerative medicine therapy into pulsed radiofrequency protocols targeting the dorsal root ganglion in patients with LLRP refractory to previous treatments represents, from an everyday clinical perspective, a highly promising and viable alternative.

## 5. Conclusions

The inclusion of ACS injections in the application of PRF for LLRP treatment is safe and accelerates improvement in pain in these patients, although without long-term improvement compared to control. Biologic treatments could be an effective and safe neuromodulator treatment for persistent radicular pain. More research is needed to understand these treatments and the influence of aspects such as application protocol.

## Figures and Tables

**Table 1:** Inclusion and exclusion criteria.

Inclusion Criteria	Exclusion Criteria
1. Patient over 18 years of age, not illiterate. 2. Unilateral, mono and/or bisegmental radicular pain of a lower extremity of at least 6 months duration. 3. If you have received treatment before, at least 3 months must have passed since the last therapy received (infiltration, radiofrequency, or surgery), and the pain should persist in the same area. 4. Submit Lumbar Magnetic Resonance Imaging (MRI), Electromyography (EMG) performed concomitantly to the pain presented by the patient at the time of inclusion in the study.	1. Refusal of the patient to participate in the study or not to sign the informed consent. 2. Allergy to intravenous iodinated contrast and/or local anesthetics. 3. Inability of the patient to maintain the prone position. 4. Systemic or local infection at the puncture site. 5. Present any of the following symptoms: atypical radiation pattern, bilateral involvement, or involvement of more than two segments or roots. 6. Concomitant pathological clinical history during the study/therapy: oncological disease, vertebral fractures, myelopathy, systemic disease, connective tissue disease, coagulation disorder, multiple sclerosis, osteomyelitis, or bone edema. 7. Pregnancy or lactation. 8. Previous treatment with placement of a spinal cord neurostimulator. 9. Previous treatment with a brain stimulator for the treatment of epilepsy or Parkinson’s disease. 10. Cardiac pacemaker carrier. 11. Patient who does not attend any of the treatment sessions for unjustified reasons.

**Table 2:** Patient characteristics according to demographic and clinical variables.

Variables		ACS, N (%)	0.9% PhS, N (%)	*p*-Value
Sex	Women	17 (50.0%)	19 (52.8%)	0.816
	Men	17 (50.0%)	17 (47.2%)	
Pain	Lumbar sciatica	2 (8.8%)	2 (5.6%)	0.933
	Radicular Pain	31 (91.2%)	34 (94.4%)	
Affected extremity	RLE	14 (41.2%)	15 (41.7%)	0.967
	LLE	20 (58.8%)	21 (58.3%)	
Major Opioid	No	32 (94.1%)	35 (97.2%)	0.522
	Yes	2 (5.9%)	1 (2.8%)	
Menor Opioid	No	26 (76.5%)	31 (86.1%)	0.300
	Yes	8 (23.5)	5 (13.9%)	
No Opioid	No	22 (64.7%)	24 (66.7%)	0.863
	Yes	12 (35.3%)	12 (33.3%)	
Previous treatment with CS infiltrations	No	0 (0.0%)	2 (5.5%)	0.130
	Yes	34 (100.0%)	34 (94.5%)	
Previous treatment with PRF	No	24 (70.6%)	29 (80.6%)	0.331
	Yes	10 (29.4%)	7 (19.4%)	
Previous treatment with surgery	No	27 (79.4%)	29 (80.6%)	0.905
	Yes	7 (20.6%)	7 (19.4%)	

ACS: Autologous Conditioned Serum. PhS: Physiological Saline. RLE: Right Lower Extremity. LLE: Left Lower Extremity. CS: Corticosteroids. PRF: Pulse Radiofrequency.

**Table 3:** Values of DN4 across the evaluated.

		30 Days		*p*-Value	3 Months		*p*-Value	6 Months		*p*-Value
		ACS N (%)	0.9% PhS N (%)		ACS N (%)	0.9% PhS N (%)		ACS N (%)	0.9% PhS N (%)	
Burning	0	12 (48.0%)	18 (69.2%)	0.163	14 (51.9%)	17 (48.6%)	0.144	9 (39.1%)	17 (60.7%)	0.300
	1	11 (44.0%)	5 (19.2%)		8 (29.6%)	7 (20.0%)		7 (30.4%)	5 (17.9%)	
	2	2 (8.0%)	3 (11.5%)		5 (18.5%)	11 (31.4%)		7 (30.4%)	6 (21.4%)	
Painful cold	0	22 (64.7%)	23 (63.9%)	0.829	21 (77.8%)	27 (77.1%)	0.930	18 (78.3%)	21 (75.0%)	0.911
	1	5 (14.7%)	7 (19.4%)		5 (18.5%)	6 (17.1%)		4 (17.4%)	5 (17.9%)	
	2	7 (20.6%)	6 (16.7%)		1 (3.7%)	2 (5.7%)		1 (4.3%)	2 (7.1%)	
Electric discharge	0	9 (29.0%)	11 (30.6%)	0.282	8 (29.6%)	9 (25.7%)	0.555	8 (34.8%)	8 (28.6%)	0.824
	1	13 (41.9%)	9 (25.0%)		11 (40.7%)	11 (31.4%)		10 (43.5%)	12 (42.9%)	
	2	9 (29.0%)	16 (44.4%)		8 (29.6%)	15 (42.9%)		5 (21.7%)	8 (28.6%)	
Tingle	0	6 (19.4%)	7 (19.4%)	0.005 **	5 (18.5%)	7 (20.6%)	0.540	4 (17.4%)	6 (21.4%)	0.915
	1	17 (54.8%)	7 (19.4%)		8 (29.6%)	6 (17.6%)		8 (34.8%)	10 (35.7%)	
	2	8 (25.8%)	22 (61.1%)		14 (51.9%)	21 (61.8%)		11 (47.8%)	12 (42.9%)	
Pricks	0	7 (22.6%)	14 (38.9%)	0.002 **	5 (18.5%)	11 (31.4%)	0.061	6 (26.1%)	11 (39.3%)	0.610
	1	18 (58.1%)	6 (16.7%)		12 (44.4%)	6 (17.1%)		5 (21.7%)	5 (17.9%)	
	2	6 (19.4%)	16 (44.4%)		10 (37.0%)	18 (51.4%)		12 (52.2%)	12 (42.9%)	
Numbness	0	9 (29.0%)	3 (8.3%)	<0.001 ***	7 (25.9%)	4 (11.4%)	0.266	8 (34.8%)	5 (17.9%)	0.385
	1	17 (54.8%)	8 (22.2%)		9 (33.3%)	17 (48.6%)		8 (34.8%)	12 (42.9%)	
	2	5 (16.1%)	25 (69.4%)		11 (40.7%)	14 (40.0%)		7 (30.4%)	11 (39.3%)	
Sting	0	25 (80.6%)	17 (47.2%)	0.002 **	21 (77.8%)	25 (71.4%)	0.792	17 (73.9%)	18 (64.3%)	0.709
	1	4 (12.9%)	3 (8.3%)		3 (11.1%)	4 (11.4%)		3 (13.0%)	4 (14.3%)	
	2	2 (6.5%)	16 (44.4%)		3 (11.1%)	6 (17.1%)		3 (13.0%)	6 (21.4%)	
Hypoesthesia to touch	0	7 (22.6%)	7 (19.4%)	0.007 **	4 (14.8%)	10 (28.6%)	0.367	5 (21.7%)	8 (28.6%)	0.763
	1	17 (54.8%)	8 (22.2%)		13 (48.1%)	12 (34.3%)		13 (56.5%)	13 (46.4%)	
	2	7 (22.6%)	21 (58.3%)		10 (37.0%)	13 (37.1%)		5 (21.7%)	7 (25.0%)	
Hypoesthesia to pinprick	0	13 (41.9%)	12 (33.3%)	0.394	15 (55.6%)	19 (54.3%)	0.174	14 (60.9%)	16 (57.1%)	0.702
	1	10 (32.3%)	9 (25.0%)		12 (44.4%)	12 (34.3%)		8 (34.8%)	9 (32.1%)	
	2	8 (25.8%)	15 (41.7%)		0 (0.0%)	4 (11.4%)		1 (4.3%)	3 (10.7%)	
Pain on rubbing	0	25 (80.6%)	21 (58.3%)	0.110	21 (77.8%)	25 (71.4%)	0.696	19 (82.6%)	21 (75.0%)	0.786
	1	4 (12.9%)	7 (19.4%)		4 (14.8%)	5 (14.3%)		2 (8.7%)	4 (14.3%)	
	2	2 (6.5%)	8 (22.2%)		2 (7.4%)	5 (14.3%)		2 (8.7%)	3 (10.7%)	

ACS: Autologous Conditioned Serum. PhS: Physiological Saline. 0: without symptoms; 1: improve symptoms; 2: worsens symptoms; ** *p* < 0.01; *** *p* < 0.001.

## Data Availability

The main investigator, in agreement with the other participating researchers, makes all data obtained in the study available with full transparency, subject to strict restrictions in accordance with patient data protection laws. In addition, the main investigator signed a commitment letter as well as a Data Confidentiality Commitment Letter, performing functions as a Clinical Record Reviewer.

## Supplementary Materials

The following supporting information can be downloaded at: https://www.mdpi.com/article/10.3390/jcm14217771/s1, Table S1: The values of both groups across the evaluated periods (baseline, 30 days, 3 months, and 6 months). ACS: Autologous Conditioned Serum. PhS: Physiological Saline. SD: standard deviation. CI: Confidence Interval. VAS: Visual Analogic Scale. DN4: *Doloeur Neurophatique* Test. MOAS: Mood Assessment Scale.

## Abbreviations

The following abbreviations are used in this manuscript:

PRF	Pulsed Radiofrequency
DRG	Dorsal Root Ganglion
ACS	Autologous Conditioned Serum
LLRP	Lower Limb Radicular Pain
PhS	Physiological Saline
NPRS	Numeric Pain Rating Scale
ODS	Oswestry Disability Scale
MOAS	Mood Assessment Scale
DN4	*Douleur Neuropathique en 4* questions
SF-12	Quality of Life assessment
CEIm	Committee Ethics Medical Investigation
DUH	Dexeus University Hospital
MRI	Magnetic Resonance Imaging
EMG	Electromyography
HIV	Human Immunodeficiency Virus
SD	Standard Deviation
REML	Restricted Maximum Likelihood
RLE	Right Lower Extremity
LLE	Left Lower Extremity
Cs	Corticosteroids
VAS	Visual Analogic Scale
CI	Confidence Interval
IL	Interleukine
FGF-2	Fibriblasts Growth Factor-2
TGF-β1	Transforming Growth Factor-β1

## References

[1] Merskey H., Bogduk N. (1994). Descriptions of Chronic Pain Syndromes and Definitions of Pain Terms.

[2] Van Boxem K., Cheng J., Patijn J., van Kleef M., Lataster A., Mekhail N., Van Zundert J. (2010). Lumbosacral radicular pain. Pain Pract..

[3] Bou Peene L., Cohen S.P., Kallewaard J.W., Wolff A., Huygen F., Gaag A.V., Monique S., Vissers K., Gilligan C., Van Zundert J. (2024). 1. Lumbosacral radicular pain. Pain Pract..

[4] Hincapié C.A., Kroismayr D., Hofstetter L., Kurmann A., Cancelliere C., Rampersaud Y.R., Boyle E., Tomlinson G.A., Jadad A.R., Hartvigsen J. (2025). Incidence of and risk factors for lumbar disc herniation with radiculopathy in adults: A systematic review. Eur. Spine J..

[5] Ostelo R.W., de Vet H.C. (2005). Clinically important outcomes in low back pain. Best Pract. Res. Clin. Rheumatol..

[6] Finnerup N.B., Kuner R., Jensen T.S. (2021). Neuropathic pain: From mechanisms to treatment. Physiol. Rev..

[7] Nijs J., Apeldoorn A., Hallegraeff H., Clark J., Smeets R., Malfliet A., Girbes E.L., De Kooning M., Ickmans K. (2015). Low back pain: Guidelines for the clinical classification of predominant neuropathic, nociceptive, or central sensitization pain. Pain Physician.

[8] Dower A., Davies M.A., Ghahreman A. (2019). Pathologic Basis of Lumbar Radicular Pain. World Neurosurg..

[9] Khorami A.K., Oliveira C.B., Maher C.G., Bindels P.J.E., Machado G.C., Pinto R.Z., Koes B.W., Chiarotto A. (2021). Recommendations for Diagnosis and Treatment of Lumbosacral Radicular Pain: A Systematic Review of Clinical Practice Guidelines. J. Clin. Med..

[10] Luijsterburg P.A., Verhagen A.P., Ostelo R.W., van Os T.A., Peul W.C., Koes B.W. (2007). Effectiveness of conservative treatments for the lumbosacral radicular syndrome: A systematic review. Eur. Spine J..

[11] Manchikanti L., Abdi S., Atluri S., Benyamin R.M., Boswell M.V., Buenaventura R.M., Bryce D.A., Burks P.A., Caraway D.L., Calodney A.K. (2013). An update of comprehensive evidence-based guidelines for interventional techniques in chronic spinal pain. Part II: Guidance and recommendations. Pain Physician.

[12] Pinto R.Z., Maher C.G., Ferreira M.L., Ferreira P.H., Hancock M., Oliveira V.C., McLachlan A.J., Koes B. (2012). Drugs for relief of pain in patients with sciatica: Systematic review and meta-analysis. BMJ.

[13] Liem L., van Dongen E., Huygen F.J., Staats P., Kramer J. (2016). The dorsal root ganglion as a therapeutic target for chronic pain. Reg. Anesth. Pain Med..

[14] Moisset X. (2024). Neuropathic pain: Evidence based recommendations. Presse Med..

[15] Finnerup N.B., Attal N., Haroutounian S., McNicol E., Baron R., Dworkin R.H., Gilron I., Haanpää M., Hansson P., Jensen T.S. (2015). Pharmacotherapy for neuropathic pain in adults: A systematic review and meta-analysis. Lancet Neurol..

[16] Moisset X., Bouhassira D., Avez Couturier J., Alchaar H., Conradi S., Delmotte M.H., Lanteri-Minet M., Lefaucheur J.P., Mick G., Piano V. (2020). Pharmacological and non-pharmacological treatments for neuropathic pain: Systematic review and French recommendations. Rev. Neurol..

[17] Abejón D., Garcia-del-Valle S., Fuentes M.L., Gómez-Arnau J.I., Reig E., van Zundert J. (2007). Pulsed radiofrequency in lumbar radicular pain: Clinical effects in various etiological groups. Pain Pract..

[18] Liem L., Russo M., Huygen F.J., Van Buyten J.P., Smet I., Verrills P., Cousins M., Brooker C., Levy R., Deer T. (2013). A multicenter, prospective trial to assess the safety and performance of the spinal modulation dorsal root ganglion neurostimulator system in the treatment of chronic pain. Neuromodulation.

[19] Munglani R. (1999). The longer-term effect of pulsed radiofrequency for neuropathic pain. Pain.

[20] Simopoulos T.T., Kraemer J., Nagda J.V., Aner M., Bajwa Z. (2008). Response to pulsed and continuous radiofrequency lesioning of the dorsal root ganglion and segmental nerves in patients with chronic lumbar radicular pain. Pain Physician.

[21] Sluijter M.E., Cosman E.R., Rittman W.B., van Kleef M. (1998). The effects of pulsed radiofrequency field applied to the dorsal root ganglion—A preliminary report. Pain Clin..

[22] Vancamp T., Levy R.M., Peña I., Pajuelo A. (2017). Relevant Anatomy, Morphology, and Implantation Techniques of the Dorsal Root Ganglia at the Lumbar Levels. Neuromodulation.

[23] Nagda J.V., Davis C.W., Bajwa Z.H., Simopoulos T.T. (2011). Retrospective review of the efficacy and safety of repeated pulsed and continuous radiofrequency lesioning of the dorsal root ganglion/segmental nerve for lumbar radicular pain. Pain Physician.

[24] Schu S., Gulve A., ElDabe S., Baranidharan G., Wolf K., Demmel W., Rasche D., Sharma M., Klase D., Jahnichen G. (2015). Spinal cord stimulation of the dorsal root ganglion for groin pain-a retrospective review. Pain Pract..

[25] Teixeira A., Grandinson M., Sluijter M. (2005). Pulsed radiofrequency for radicular pain due to a herniated intervertebral disc—An initial report. Pain Pract..

[26] Tortora F., Negro A., Russo C., Cirillo S., Caranci F. (2021). Chronic intractable lumbosacral radicular pain, is there a remedy? Pulsed radiofrequency treatment and volumetric modifications of the lumbar dorsal root ganglia. Radiol. Med..

[27] Van Boxem K., Huntoon M., Van Zundert J., Patijn J., van Kleef M., Joosten E.A. (2014). Pulsed radiofrequency: A review of the basic science as applied to the pathophysiology of radicular pain: A call for clinical translation. Reg. Anesth. Pain Med..

[28] Park S., Park J.H., Jang J.N., Choi S.I., Song Y., Kim Y.U., Park S. (2024). Pulsed radiofrequency of lumbar dorsal root ganglion for lumbar radicular pain: A systematic review and meta-analysis. Pain Pract..

[29] Shakouri S.K., Dolati S., Santhakumar J., Thakor A.S., Yarani R. (2021). Autologous conditioned serum for degenerative diseases and prospects. Growth Factors.

[30] Auw Yang K.G., Raijmakers N.J., van Arkel E.R., Caron J.J., Rijk P.C., Willems W.J., Zijl J.A., Verbout A.J., Dhert W.J., Saris D.B. (2008). Autologous interleukin-1 receptor antagonist improves function and symptoms in osteoarthritis when compared to placebo in a prospective randomized controlled trial. Osteoarthr. Cartil..

[31] Baltzer A.W., Moser C., Jansen S.A., Krauspe R. (2009). Autologous conditioned serum (Orthokine) is an effective treatment for knee osteoarthritis. Osteoarthr. Cartil..

[32] Baltzer A.W., Ostapczuk M.S., Stosch D., Seidel F., Granrath M. (2013). A new treatment for hip osteoarthritis: Clinical evidence for the efficacy of autologous conditioned serum. Orthop. Rev..

[33] Baselga J., Hernandez P.M. (2013). ORTHOKINE-Therapy for High-Pain Knee Osteoarthritis (OA) May Delay Surgery. Independent 2-Year Case Follow-Up.

[34] Darabos N., Haspl M., Moser C., Darabos A., Bartolek D., Groenemeyer D. (2011). Intraarticular application of autologous conditioned serum (ACS) reduces bone tunnel widening after ACL reconstructive surgery in a randomized controlled trial. Knee Surg. Sports Traumatol. Arthrosc..

[35] Manchikanti L., Alturi S., Sanapati M., Hirsch J.A., Manchikanti L., Navani A., Sanapati M. (2024). Epidural Administration of Biologics. Essentials of Regenerative Medicine in Interventional Pain Management.

[36] Wright-Carpenter T., Klein P., Schäferhoff P., Appell H.J., Mir L.M., Wehling P. (2004). Treatment of muscle injuries by local administration of autologous conditioned serum: A pilot study on sportsmen with muscle strains. Int. J. Sports Med..

[37] Chan A.-W., Tetzlaff J.M., Gøtzsche P.C., Altman D.G., Mann H., Berlin J., Dickersin K., Hróbjartsson A., Schulz K.F., Parulekar W.R. (2013). *SPIRIT* 2013 Explanation and Elaboration: Guidance for protocols of clinical trials. BMJ.

[38] Butcher N.J., Monsour A., Mew E.J., Chan A.W., Moher D., Mayo-Wilson E., Terwee C.B., Chee-A-Tow A., Baba A., Gavin F. (2022). Guidelines for Reporting Outcomes in Trial Reports: The CONSORT-Outcomes 2022 Extension. JAMA.

[39] Homs M., Milà R., Valdés R., Blay D., Borràs R.M., Parés D. (2023). Efficacy of conditioned autologous serum therapy (Orthokine^®^) on the dorsal root ganglion in patients with chronic radiculalgia: Study protocol for a prospective randomized placebo-controlled double-blind clinical trial (RADISAC trial). Trials.

[40] Childs J.D., Piva S.R., Fritz J.M. (2005). Responsiveness of the numeric pain rating scale in patients with low back pain. Spine (Phila Pa 1976).

[41] Garg A., Pathak H., Churyukanov M.V., Uppin R.B., Slobodin T.M. (2020). Low back pain: Critical assessment of various scales. Eur. Spine.

[42] Streiner D.L., Norman G.R. (2008). Health Measurement Scales: A Practical Guide to Their Development and Use.

[43] Fairbank J.C., Pynsent P.B. (2000). The Oswestry Disability Index. Spine (Phila Pa 1976).

[44] Sanz J. (2001). Un instrumento para evaluar la eficacia de los procedimientos de inducción de estado de ánimo: “La escala de valoración del estado de ánimo” (EVEA). Anál. Modif. Conducta.

[45] Gandek B., Ware J.E., Aaronson N.K., Apolone G., Bjorner J.B., Brazier J.E., Bullinger M., Kaasa S., Leplege A., Prieto L. (1998). Cross-validation of item selection and scoring for the SF-12 Health Survey in nine countries: Results from the IQOLA Project. J. Clin. Epidemiol..

[46] Vilagut G., Valderas J.M., Ferrer M., Garin O., López-García E., Alonso J. (2008). Interpretación de los cuestionarios de salud SF-36 y SF-12 en España: Componentes físico y mental [Interpretation of SF-36 and SF-12 questionnaires in Spain: Physical and mental components]. Med. Clin..

[47] Attal N., Perrot S., Fermanian J., Bouhassira D. (2011). The neuropathic components of chronic low back pain: A prospective multicenter study using the DN4 Questionnaire. J. Pain.

[48] Perez C., Galvez R., Huelbes S., Insausti J., Bouhassira D., Diaz S., Rejas J. (2007). Validity and reliability of the Spanish version of the DN4 (Douleur Neuropathique 4 questions) questionnaire for differential diagnosis of pain syndromes associated to a neuropathic or somatic component. Health Qual. Life Outcomes.

[49] Becker C., Heidersdorf S., Drewlo S., de Rodriguez S.Z., Krämer J., Willburger R.E. (2007). Efficacy of epidural perineural injections with autologous conditioned serum for lumbar radicular compression: An investigator-initiated, prospective, double-blind, reference-controlled study. Spine (Phila Pa 1976).

[50] Ruiz-Lopez R., Tsai Y.C. (2020). A randomized double-blind controlled pilot study comparing leucocyte-rich platelet-rich plasma and corticosteroid in caudal epidural injection for complex chronic degenerative spinal pain. Pain Pract..

[51] Godek P. (2016). Use of autologous serum in treatment of lumbar radiculopathy pain: Pilot study. Ortop. Traumatol. Rehabil..

[52] Ravi Kumar H.S., Goni V.G., Batra Y.K. (2015). Autologous conditioned serum as a novel alternative option in the treatment of unilateral lumbar radiculopathy: A prospective study. Asian Spine J..

[53] Goni V.G., Singh Jhala S., Gopinathan N.R., Behera P., Batra Y.K., Arjun R.H.H.A., Guled U., Vardhan H. (2015). Efficacy of epidural perineural injection of autologous conditioned serum in unilateral cervical radiculopathy: A pilot study. Spine (Phila Pa 1976).

[54] Aghamohammadi D., Sharifi S., Shakouri S.K., Eslampour Y., Dolatkhah N. (2022). Autologous conditioned serum (Orthokine) injection for treatment of classical trigeminal neuralgia: Results of a single-center case series. J. Med. Case Rep..

[55] Hassanien M., Elawamy A., Kamel E.Z., Khalifa W.A., Abolfadl G.M., Roushdy A.S.I., El Zohne R.A., Makarem Y.S. (2020). Perineural platelet-rich plasma for diabetic neuropathic pain: Could it make a difference?. Pain Med..

[56] Anitua E., Troya M., Alkhraisat M.H. (2024). Effectiveness of platelet derivatives in neuropathic pain management: A systematic review. Biomed. Pharmacother..

[57] Sánchez M., Garate A., Delgado D., Padilla S. (2017). Platelet-rich plasma, an adjuvant biological therapy to assist peripheral nerve repair. Neural Regen. Res..

[58] Anjayani S., Wirohadidjojo Y.W., Adam A.M., Suwandi D., Seweng A., Amiruddin M.D. (2014). Sensory improvement of leprosy peripheral neuropathy in patients treated with perineural injection of platelet-rich plasma. Int. J. Dermatol..

[59] Sánchez M., Yoshioka T., Ortega M., Delgado D., Anitua E. (2014). Ultrasound-guided platelet-rich plasma injections for the treatment of common peroneal nerve palsy associated with multiple ligament injuries of the knee. Knee Surg. Sports Traumatol. Arthrosc..

[60] Kuffler D. (2013). Platelet-rich plasma and the elimination of neuropathic pain. Mol. Neurobiol..

[61] Gupta S., Paliczak A., Delgado D. (2020). Evidence-based indications of platelet-rich plasma therapy. Expert Rev. Hematol..

[62] Frisbie D.D., Kawcak C.E., Werpy N.M., Park R.D., Mc-Ilwraith C.W. (2007). Clinical, biochemical, and histologic effects of intra-articular administration of autologous conditioned serum in horses with experimentally induced osteoarthritis. Am. J. Vet Res..

[63] Wehling P., Moser C., Frisbie D., McIlwraith C.W., Kawcak C.E., Krauspe R., Reinecke J.A. (2007). Autologous conditioned serum in the treatment of orthopedic diseases: The orthokine therapy. BioDrugs.

[64] Hung A.L., Lim M., Doshi T.L. (2017). Targeting cytokines for treatment of neuropathic pain. Scand. J. Pain..

[65] Desai M.J., Mansfield J.T., Robinson D.M., Miller B.C., Borg-Stein J. (2020). Regenerative Medicine for Axial and Radicular Spine-Related Pain: A Narrative Review. Pain Pract..

[66] Kawabata S., Akeda K., Yamada J., Takegami N., Fujiwara T., Fujita N., Sudo A. (2023). Advances in Platelet-Rich Plasma Treatment for Spinal Diseases: A Systematic Review. Int. J. Mol. Sci..

[67] Kubrova E., Martinez Alvarez G.A., Her Y.F., Pagan-Rosado R., Qu W., D’Souza R.S. (2022). Platelet Rich Plasma and platelet-related products in the treatment of radiculopathy—A systematic review of the literature. Biomedicines.

[68] de Moraes Ferreira Jorge D., Huber S.C., Rodrigues B.L., Da Fonseca L.F., Azzini G.O.M., Parada C., Paulus-Romero C., Lana J.F.S.D. (2022). The mechanism of action between pulsed radiofrequency and orthobiologics: ¿Is there a synergistic effect?. Int. J. Mol. Sci..

