# Supplementary material for: Efficacy of Autologous Conditioned Serum on the Dorsal Root Ganglion in Patients with Chronic Radicular Pain: Prospective Randomized Placebo-Controlled Double Blind Clinical Trial (RADISAC Trial)
Source: J Clin Med. 2025 Nov 1;14(21):7771. doi: 10.3390/jcm14217771 (PMC12608376; doi:10.3390/jcm14217771)
Supplement: Supplementary file 1 [file jcm-14-07771-s001.zip › jcm-3941511-supplementary.pdf]

Table S1. The values of both groups across the evaluated periods (baseline, 30 days, 3 months, and 6 months). ACS: Autologous Conditioned Serum. PhS: Physiological Saline. SD: standard deviation. CI: Confidence Interval. NPRS: Numeric Pain Rating Scale. DN4: Doloeur Neurophatique Test. MOAS: Mood Assessment Scale.

Table 3. The values of both groups across the evaluated periods (baseline, 30 days, 3 months, and 6 months)

Scale	Baseline						1 month						3 months						6 months						P-value a	P-value b	P-value c
	ACS			0.9% PhS			ACS			0.9% PhS			ACS			0.9% PhS			ACS			0'9% PhS					
	N	Mean (SD)	CI95%	N	Mean (SD)	CI95%	N	Mean (SD)	CI95%	N	Mean (SD)	CI95%	N	Mean (SD)	CI95%	N	Mean (SD)	CI95%	N	Mean (SD)	CI95%	N	Mean	CI95%			
**NPRS**	34	8.15 (1.37)	[7.67;8.63]	36	7.61 (1.02)	[7.27;7.96]	31	3.16 (2.02)	[2.42;3.90]	36	6.64 (2.37)	[5.84;7.44]	27	3.81 (2.13)	[2.97;4.66]	35	4.91 (3.07)	[3.86;5.97]	23	4.35 (2.79)	[3.14;5.55]	28	4.11 (3.31)	[2.82;5.39]	0.194	0.003	<0.001 ***
**DN4**	34	5.15 (1.73)	[4.54;5.75]	36	5.17 (2.49)	[4.32;6.01]	31	4.39 (2.25)	[3.56;5.21]	36	3.61 (2.43)	[2.79;4.43]	27	2.37 (1.98)	[1.59;3.16]	35	3.11 (2.83)	[2.14;4.09]	23	2.30 (1.89)	[1.49;3.12]	28	2.50 (2.70)	[1.45;3.55]	0.903	0.292	0.066
**MOAS depression**	34	16.26 (9.50)	[12.95;19.58]	36	16.03 (11.1)	[12.27;19.79]	31	14.55 (9.45)	[11.08;18.02]	36	14.11 (11.6)	[10.17;18.06]	27	12.00 (8.97)	[8.45;15.55]	35	11.94 (12.2)	[7.75;16.14]	23	13.83 (9.19)	[9.85;17.80]	28	10.64 (10.0)	[6.73;14.55]	0.645	0.003	0.603
**MOAS anxiety**	34	20.76 (9.59)	[17.42;24.11]	36	20.92 (16.6)	[15.28;26.56]	31	18.35 (10.5)	[14.47;22.24]	36	15.92 (10.8)	[12.24;19.59]	27	15.00 (9.39)	[11.28;18.72]	35	14.77 (11.8)	[10.70;18.85]	23	17.43 (10.8)	[12.74;22.13]	28	13.11 (11.2)	[8.76;17.45]	0.652	<0.001 ***	0.211
**MOAS anger**	34	18.12 (10.3)	[14.52;21.72]	36	16.06 (12.5)	[11.82;20.29]	31	13.48 (11.7)	[9.19;17.78]	36	13.61 (12.2)	[9.45;17.77]	27	12.30 (10.4)	[8.15;16.44]	35	13.40 (12.9)	[8.96;17.84]	23	14.57 (11.5)	[9.59;19.54]	28	11.79 (11.9)	[7.16;16.41]	0.634	0.014*	0.679
**MOAS joy**	34	16.82 (7.97)	[14.04;19.60]	36	20.78 (8.74)	[17.82;23.74]	31	20.48 (8.62)	[17.32;23.65]	36	22.56 (9.43)	[19.36;25.75]	27	21.85 (7.72)	[18.80;24.91]	35	21.17 (10.6)	[17.53;24.81]	23	19.87 (8.24)	[16.31;23.43]	28	23.25 (9.50)	[19.57;26.93]	0.160	0.006**	0.281
**Oswestry**	34	23,41 (10,4)	[19,75;27,07]	36	21,47 (10,4)	[17,92;25,02]	30	14,83 (8,91)	[11,5;18,16]	33	14,51 (9,07)	[11,29;17,73]	25	13,88 (8,46)	[10,38;17,37]	32	14,12 (9,89)	[10,55;17,69]	22	14,04 (9,46)	[9,84;18,24]	28	13,64 (10,4)	[9,57;17,7]	0.633	<0.001 ***	0.676

*ACS: Autologous Conditioned Serum. PhS: Physiological Saline. SD: standard deviation. CI: Confidence Interval. NPRS: Numeric Pain Rating Scale . DN4: Doloeur Neurophatique Test. MOAS: Mood Assessment Scale. [a]P-value between groups; [b]P-value within groups; [c]P-value time x Group. * $p < 0.05$; ** $p < 0.01$; *** $p < 0.001$*
